# The Impact of the Built Environment and Social Environment on Physical Activity: A Scoping Review

**DOI:** 10.3390/ijerph20126189

**Published:** 2023-06-20

**Authors:** Yiyu Wang, Bert Steenbergen, Erwin van der Krabben, Henk-Jan Kooij, Kevin Raaphorst, Remco Hoekman

**Affiliations:** 1 Behavioral Science Institute, Radboud University, 6525 XZ Nijmegen, The Netherlands; bert.steenbergen@ru.nl; 2 Department of Geography, Planning, and Environment, Institute for Management Research, Radboud University, 6525 XZ Nijmegen, The Netherlands; erwin.vanderkrabben@ru.nl (E.v.d.K.); henk-jan.kooij@ru.nl (H.-J.K.); kevin.raaphorst@ru.nl (K.R.); 3 Mulier Institute, 3584 AA Utrecht, The Netherlands; r.hoekman@mulierinstituut.nl

**Keywords:** physical activity, built environment, social environment

## Abstract

This scoping review aims to provide an overview of the current state of physical activity research, focusing on the interplay between built and social environments and their respective influences on physical activity. We comprehensively searched electronic databases to identify relevant studies published between 2000 and 2022. A total of 35 articles have been reviewed based on the research question. The review found that built and social environments influence physical activity, and consideration of people’s perceptions of their surroundings can provide further insight. The literature was summarized, and recommendations were made for future research. Findings suggest that interventions targeting built and social environments can promote physical activity effectively. However, limitations in the literature exist, including a need for more standardization in research methods and consistency in measurement tools.

## 1. Introduction

Moderate-to-vigorous physical activity (PA) is critical to reducing chronic diseases such as obesity and enhancing the population’s health. Although physical activity has raised more attention in the past years, public health statistics still show that, globally, 28% of adults aged 18 and over were not active enough to reach the WHO’s recommendation in 2016 (men, 23%; women, 32%) [1]. This growing development of insufficient physical activity has, in turn, exacerbated obesity, cardiovascular disease, diabetes, and other chronic diseases. These chronic diseases account for approximately 70% of deaths globally [1]. In the context of inactivity levels and the recent COVID-19 pandemic, research on physical activity and how to promote it is of great importance.

One of the many factors for improving physical activity is the built environment. The built environment is essential to the physical environment, including urban and architectural design, land use, transportation, and corresponding infrastructure support facilities [2]. For example, studies show that mixed-use residential, commercial, office, entertainment, and other land use can significantly increase walking [3]. In addition, the street network pattern can influence the choice of travel routes and modes of transportation. For example, high connectivity encourages active transportation by reducing travel distances while providing multiple travel route choices [4,5]. Furthermore, good accessibility and connectivity of destinations are beneficial in increasing opportunities for people to walk/bike commute or travel daily [6]. Based on the existing research on the built environment and physical activity and the evidence from this research, the built environment has become a critical intervention and policy tool promoting physical activity.

Relevant policies involving urban planning and public transportation are closely related to the level of participation of the public in physical activities. In urban planning, for the management of land-use types, relevant laws and policies can be formulated to require that a certain amount and proportion of land be used for the construction of physical activity facilities to ensure that sufficient venues are provided for people’s physical activities [7]. For transport intervention, paving sidewalks and bicycle lanes in cities will reduce urban traffic congestion and environmental pollution caused by car exhaust and help more people choose physical activities such as walking and cycling [8]. However, it is challenging to predict physical activity patterns in the built environment simply by the friendliness of the built environment. Individual physical activity requires not only the support of the built environment but also the support of the social environment.

The social environment includes individual factors (including age, gender, fitness, or biology such as genetic predispositions and neurological development, etc.), social networks (including family, peers, school, community, and work), and a wide range of background conditions (such as culture and economy) [9]. Several studies have examined the relationship between critical demographic variables such as gender, income level, education level, and physical activity. Some research states that men are more involved in physical activities than women, particularly in high-income countries [10,11,12,13]. In addition, cultural norms around gender roles and expectations can also influence women’s engagement in physical activity, particularly in more conservative societies [14]. Others have reported that in some African countries, such as Mozambique, Tanzania, and Uganda, women tend to engage in more physical activity than men [15]. Studies have found that in Asian countries, such as China, women tend to engage in more physical activity than men [16]. In addition, individuals with higher income levels [17,18] and higher education levels are more likely to participate in leisure-time physical activities [19,20].

Research on the factors that affect physical activity individuals has received increased attention in urban planning, sociology, and behavior research. With the introduction of the social ecological model of health behavior [21], research on the influencing factors of behavior is no longer limited to the individual level of age, gender, knowledge, and skills. Instead, it has become multifactorial, also including aspects of work, the built environment, and leisure time. Its core feature is that physical activity results from multiple factors [22]. Bronfenbrenner’s social ecological theoretical model provides a comprehensive and interdisciplinary analysis framework, broadens the research ideas of physical activity-related factor analysis, and has been widely used in empirical research. In addition, it exemplifies the urban built environment’s influence on physical activity [23,24].

However, compared to the abundance of research that uses the social ecological model to study the influence of the built environment on physical activity, research on the intricate relation between built and social environments and their combined influence on physical activity is relatively scarce. As a notable exception, a study in Hong Kong takes housing type as an indicator and found that this aspect of the built environment is a significant determinant of social environments, social contacts, and activity-travel behavior [25]. Similar research in the US also found that housing type limits options for a healthy lifestyle and social cohesion, which in turn influences physical activity [26]. From the perspective of research content, there is an excellent potential for research on the relationship between built and social environmental factors that affect physical activity and types of physical activity. This necessitates the systematic exploration of physical activity research by integrating the urban planning discipline with sociology and behavioral approaches.

We focus on filling this knowledge gap in the present scoping review. Specifically, we examine the state of the art in physical activity research, which includes the interaction between the built environment and the social environment and the advantages of combining built and social environments and the impact of this combination on physical activity research. This review provides a solid base and lays bare areas of future research that combine the abovementioned disciplines. Finally, we discuss the results in light of the potential advantages of interdisciplinary approaches for promoting physical activity.

## 2. Methods

**Figure 1:**
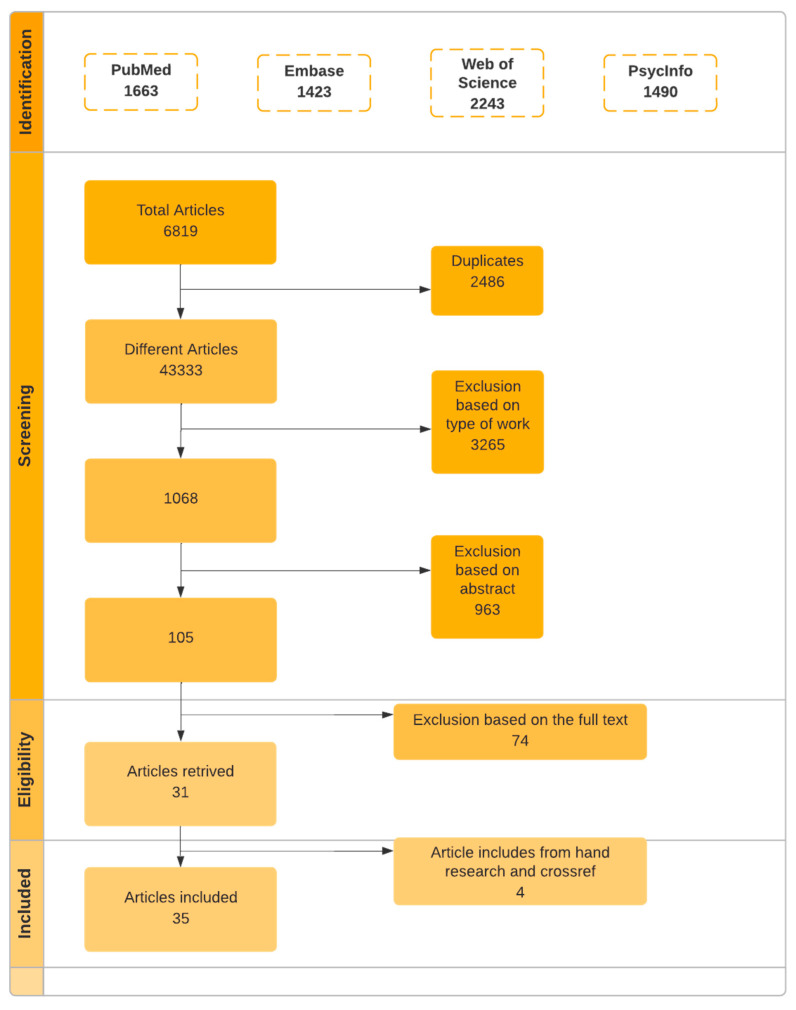
The data extraction process.

The research adopts the [27] scoping review research framework, divided into five steps: (1) Clarify the research question; (2) Determine the relevant research by searching PubMed, EMBASE, PsycINFO, and Web of Science databases; (3) Screen the target literature; (4) Data extraction, i.e., according to the research purpose, develop an information extraction table to incorporate critical research information, including basic information such as published author, year, and country, as well as research process information such as research purpose, research design, data extraction method, and sampling method. Relevant information also includes research results such as outcome measurement, methods, and leading research results; and (5) summarize and present the results. This research uses thematic analysis to classify the themes mentioned above and presents findings as tables and summary paragraphs. The following graphic describes the procedure of data retrieval, including the number of articles retrieved in each stage of the process. See Figure 1. The data extraction process.

### 2.1. The Research Questions

Based on the questions addressed in the introduction, this scoping review defines the focused research questions as follows:

(1) To what extent has current research included both the built environment and the social environment as factors that impact physical activity?
(2) What can we learn from this research concerning the advantages of the combination of built and social environments and the impact of this combination on physical activity?

### 2.2. Searching for Relevant Research

Literature retrieval and reporting methods follow the requirements of scoping reviews. An information specialist of the library was consulted for the evaluation setup, which included choosing the right databases, suitable keywords, and mesh terms. Literature data were collected through Web of Science, PubMed, EMBASE, PsycInfo database, and Google Scholar. The three primary keywords were the built environment, social environment, and physical activity.

#### 2.2.1. Physical Activity

Physical activity mainly refers to any physical actions that require energy consumption produced by skeletal muscles, including work, housework, transportation, leisure, and other activities [28]. The measurement of physical activity level mainly adopts self-reporting methods and, to a lesser extent, physical activity trackers. Physical activity outcomes include traffic-related walking, recreational walking, cycling, moderate-to-vigorous-intensity physical activity (MVPA), and total physical activity.

#### 2.2.2. Built Environment

The built environment is an essential part of the physical environment, including urban and architectural design, land use, transportation, and corresponding infrastructure support facilities. The built environment is defined as the objective and subjective characteristics of the physical context in which people spend their time (e.g., home, neighborhood), including aspects of urban design, (e.g., presence of sidewalks), traffic density and speed, distance to and design of venues for physical activity (PA) (e.g., parks), and crime and safety [29].

#### 2.2.3. Social Environment

According to the social ecological model, human health is determined by individual factors (including age, gender, fitness, or biology such as genetic predispositions and neurological development, etc.), social networks (family, peers, school, community, work), environment (built environment, social environment, policy), and a wide range of background conditions (such as culture and economy) [9]. The social environment refers to the relationships, culture, and society that individuals interact with, including social influencers such as friends and family [30].

#### 2.2.4. Search Strategy

An information specialist performed the search on 30 March 2020, and the search formula comprised the main search terms (topic terms) connected by logical words “AND” and “OR”. For example, on PubMed, the keywords and mesh terms that were chosen are: ((Exercise [MesH] OR Bicycling [MesH] OR Sedentary behavior [MesH] OR physical activit* [tiab] OR exercise* [tiab] OR walking [tiab] OR bicycling [tiab] OR cycling [tiab] OR sedentary behavior [tiab] OR sedentary behaviour [tiab]) AND (Environment Design [MesH] OR environment design* [tiab] OR Urban planning [tiab] OR urban environment* [tiab] OR built environment* [tiab] OR physical environment* [tiab] OR healthy place* [tiab] OR universal design* [tiab] OR human centered design* [tiab] OR design for all [tiab])) AND (Social environment [MesH] OR social environment* [tiab] OR social context* [tiab] OR social ecology* [tiab] OR community network* [tiab] OR community health network* [tiab] OR social support [tiab]). See the search strategy for EMBASE, PsycInfo, and Web of science in Note 1.

Note 1: Search strategy

(1) PubMed

#1 Physical activity/Walking/Cycling

Exercise [MesH] OR Bicycling [MesH] OR Sports [MesH] OR Sedentary behavior [MesH] OR physical activit* [tiab] OR exercise* [tiab] OR walking [tiab] OR bicycling [tiab] OR cycling [tiab] OR sedentary behavior [tiab] OR sedentary behaviour [tiab] OR sport* [tiab]

#2 Built Environment—urban planning intervention to promote active living at street level, community/neighborhood level

Environment Design [MesH] OR environment design* [tiab] OR Urban planning [tiab] OR urban environment* [tiab] OR built environment* [tiab] OR physical environment* [tiab] OR safety [tiab] OR healthy place* [tiab] OR universal design* [tiab] OR human centered design* [tiab] OR design for all [tiab] OR land use [tiab] OR land usage [tiab]

#3 Social Environment

Social environment [MesH] OR Interpersonal relations [MesH] OR social environment* [tiab] OR social context* [tiab] OR social ecology* [tiab] OR community network* [tiab] OR community health network* [tiab] OR social support [tiab] OR safety [tiab] OR social relation* [tiab] OR interpersonal relation* [tiab]

(2) Embase

#1 Physical activity/Walking/Cycling

Exp exercise/OR Exp physical activity/OR exp sports/OR exp sedentary lifestyle/OR (physical activit* OR exercise* OR walking OR bicycling OR cycling OR sedentary behavior OR sedentary behavior OR sport*).ti,ab,kw.

#2 Built Environment—urban planning intervention to promote active living at street level, community/neighborhood level

Exp environmental planning/OR (environment design* OR Urban planning OR urban environment* OR built environment* OR physical environment* OR healthy place* OR universal design* OR human centered design* OR design for all OR land usage).ti,ab,kw.

#3 Social Environment

Exp social environment/OR (social environment* OR social context* OR social ecology* OR community network* OR community health network* OR social support safety OR social relation* OR interpersonal relation*).ti,ab,kw.

(3) PsycInfo

#1 Physical activity/Walking/Cycling

Exp exercise/OR Exp physical activity/OR exp sports/OR exp sedentary lifestyle/OR (physical activit* OR exercise* OR walking OR bicycling OR cycling OR sedentary behavior OR sedentary behavior OR sport*).ti,ab,id.

#2 Built Environment—urban planning intervention to promote active living at street level, community/neighborhood level

Exp environmental planning/OR (environment design* OR Urban planning OR urban environment* OR built environment* OR physical environment* OR healthy place* OR universal design* OR human centered design* OR design for all OR land usage).ti,ab,id.

#3 Social Environment

Exp social environment/OR (social environment* OR social context* OR social ecology* OR community network* OR community health network* OR social support safety OR social relation* OR interpersonal relation*).ti,ab,id.

(4) Web of Science

#1 Physical activity/Walking/Cycling

TOPIC: (“physical activit*” OR exercise* OR walking OR bicycling OR cycling OR “sedentary behavior” OR “sedentary behaviour” OR sport*)

#2 Built Environment—urban planning intervention to promote active living at street level, community/neighborhood level

TOPIC: (“environment design*” OR “Urban planning” OR “urban environment*” OR “built environment*” OR “physical environment*” OR “healthy place*” OR “universal design*” OR “human centered design*” OR “design for all” OR “land use” OR “land usage”)

#3 Social Environment

TOPIC: (“social environment*” OR “social context*” OR “social ecology*” OR “community network*” OR “community health network*” OR “social support” OR “safety” OR “social relation” OR “interpersonal relation”)

### 2.3. Screening the Target Literature

Literature screening was performed in three consecutive steps: (1) remove duplications, (2) screen the article’s abstract, and (3) set criteria for eligible articles.

The criteria were set based on the research questions. To answer the research questions, the selected article was required to contain three key concepts, which are physical activity, the built environment, and the social environment.

After discussion with the team members, the criteria of the eligible articles were set as follows: (1) includes objective built environment measurements; (2) includes social environment measurements; (3) includes physical activity measurements such as traffic-related walking, recreational walking, cycling, moderate-to-vigorous-intensity physical activity (MVPA), and total physical activity; (4) includes a human population.

The criteria for non-eligible articles were set as follows: (1) only includes an analysis of the built environment’s impact on physical activity; (2) only includes an analysis of the impact of the social environment on physical activity; (3) only involves disease-related physical activity; (4) does not perform in the real world; (5) does not include objective environment measurement; (6) does not include physical activity.

### 2.4. Data Extraction

According to the research purpose, the research team created a data extraction table that includes main characteristics incorporating the selected articles’ critical information. Data extraction includes the following: (1) research aim, (2) physical activity measurements, (3) type of physical activity, (4) built environment measurements, (5) social environment measurements, (6) data sample, (7) population demographic, (8) location, (9) analytical method, and (10) findings.

### 2.5. Summarizing and Presenting the Results

To summarize the results, this research used thematic analysis to classify the abovementioned themes and report them in tables and summary paragraphs. First, we created a table that provides an overview of the characteristics of the selected articles (Table A1). Second, we focused on the research combining the built and social environments’ impacts on physical activities. Finally, we discussed the results and provided suggestions for future studies.

## 3. Results

### 3.1. Overview of the Studies—Table A1 Provides an Overview of the Characteristics of the Selected Studies (See Appendix A)

#### 3.1.1. Demographic Characteristics

The 35 selected studies showed a great variety of research populations. Of the 35 included studies, 22 studies had adults as participants (n = 63%). Thirteen studies (n = 35.37%) had adolescents as participants. Eight studies focused on females of different ethnic minority groups (n = 23%). The final three studies were conducted on the elderly population (n = 8%).

#### 3.1.2. Geographic Characteristics

The selected studies covered geographic diversification. Thirteen studies were conducted in the US (n = 37%). A further 10 studies were conducted in European countries (n = 28.5%) and 6 studies were performed in Australia (n = 17%). Finally, three studies from Asian countries (n = 8%) and two from Brazil (n = 5%) were found.

#### 3.1.3. Social Environment Characteristics

Studies examined various aspects of the social environment. At the individual level, demographic characteristics were evaluated, and SES (socioeconomic status) is one of the most evaluated characteristics. Of the 35 studies, 30 studies evaluated the SES of the participants (n = 86%), and 5 of the 30 studies had a research focus on low-SES communities. At the community level, the most evaluated factor is community/neighborhood safety (14/35). In addition, general social support (10/35) is given much attention. Finally, social cohesion (8/35) has also been highlighted.

#### 3.1.4. Built Environment Characteristics

Concerning aspects of the built environment, of the total 35 studies, the majority of articles (n = 30.85%) focused on community. Regarding more specific urban settings, five studies (n = 15%) looked into the school environment. Researchers have evaluated various built environment factors in the papers that we have reviewed. Accessibility (20/35) and connectivity (18/35) have received the most attention, while other studies have also incorporated environment perception into evaluation of the built environment (15/35).

#### 3.1.5. Physical Activity Characteristics

Physical activity was divided into commuting, leisure, and total physical activity. For commuting-related physical activity, four studies (n = 35.11%) measured the daily commutes (walking or cycling) to transportation hubs and final destinations. Twenty-six studies (n = 75%) focused on leisure-time physical activity estimates, such as minutes of moderate-to-vigorous physical activity per day or week. The remaining five studies (n = 14%) reported the total physical activity in minutes.

#### 3.1.6. Measurement Characteristics

All 35 selected studies measured the perception of the social environment through surveys/questionnaires/interviews. These methods were different for the built environment. Of the 35 studies, 11 (n = 31%) used GIS (geographic information systems) to provide an objective measurement of aspects of the built environment. Fourteen studies (n = 40%) used surveys/questionnaires/interviews regarding perceptions of the built environment to represent the subjective measure of the physical environment in which participants lived. Children’s perception of the neighborhood was measured with the help of their parents. One study used mapping exercises to determine the perception of a neighborhood. Here, participants were asked to use colored pens to identify key routes and destinations related to their walking activities [31]. Among the 35 studies, the physical activity data of 29 studies were obtained through self-reported questionnaires. Six studies applied physical activity tracking devices to participants. Four of the latter six studies combined questionnaires and direct activity monitoring.

### 3.2. Social Environmental Impact on Physical Activity

#### 3.2.1. Interpersonal Level

At the interpersonal level, participation and persistence are essential for physical activity, and social support is an important variable that promotes people’s participation in physical activity. Individual social support generally comes from family and peer support. Three of thirty-five studies showed that support for physical activity from closely related persons positively impacts an individual’s physical activity behavior at all ages [32,33,34]. In three studies with adolescents, it was shown that family, friends, and peers are the sources of social support affecting participation in physical activity [31,35,36]. Notably, in two studies with children, participation in physical activity, occupation, and education level of parents and other family members were used as independent variables to examine children’s physical activity [36,37]. Social interactions were also considered vital in two studies, one with children and adolescents [33] and another with adults [36]. Children and adolescents who hang out and play with their friends are more prone to physical activity during the weekdays [33,36]. One study also suggested that physical activity role models promote people’s participation in physical activity [38].

#### 3.2.2. Community Level

At the community level, the safety of the community has been studied. Five studies used local crime rates to determine safety [38] and four studies applied perceived neighborhood safety surveys [33,39,40,41]. One study found that people are more engaged in physical activity if the community’s safety can be guaranteed. Conversely, if people’s perceived community environment is unsafe, it will lead to decreased physical activity, a decline in health and fitness levels, and social isolation [42]. In addition, social cohesion was mentioned in eight studies. This aspect is used to examine the sense of belonging [43], trust [44], value [43], and healthy beliefs [45]. Moreover, social norms can also change people’s physical activity and health behaviors. Social norms reflect standards of behavior and generally accepted values in society. For example, one study’s results showed that if it is common in a neighborhood for people to walk, then people in the neighborhood are more likely to walk to a travel destination [43]. In studies with female participants, the results also indicated that seeing people exercise in the neighborhood was positively related to higher levels of physical activity [43,46,47,48,49].

#### 3.2.3. Other Aspects

In addition to the aspects mentioned above, three studies from Australia examined the ownership of sports membership cards [22,32,45], and one study looked into dog ownership [50]. The study provided evidence that the ownership of dogs positively impacted an individual’s physical activity level. Another two studies showed a lack of positive impact of sports membership on adults’ physical activity [22,32]. Finally, one study from the US showed that participation in a recreational program with friends was associated with an increased likelihood of the elderly’s physical activity [44].

### 3.3. Built Environmental Impact on Physical Activity

Researchers examined a variety of built environmental features in the selected studies.

#### 3.3.1. Accessibility

Three studies have pointed out that objective accessibility between residences and buses or subway stations is significantly correlated to an increase in the daily walking/cycling commuting of residents [36,51,52]. In addition, three studies on adolescents [35,39,53] found that the closer the a destination (including parks, workplaces, and commercial facilities), the greater the opportunity for transformational walking among adolescents. Similar to objective accessibility, subjectively perceived accessibility is positively correlated with residents’ physical activity level. Six studies showed that the better the perceived distance of residents to public transport stops or destinations, the more likely they are to increase walking or cycling opportunities [42,43,46,47,48,49,54] and recreational physical activity frequency [39].

#### 3.3.2. Connectivity

Five studies primarily examined the relationship between road network connectivity and walking or cycling behavior by measuring neighborhood street length, area, intersection density, street density, and other indicators [22,32,36,50]. Nine studies showed that the shorter the distance to a destination, the more likely it is that commuting via walking or cycling to that destination is increased in adults or children [31,42,43,46,47,48,49,55]. One study also showed this for recreational walking and commuting via walking in an elderly population [56]. In addition, three studies suggested that the better adults perceive neighborhood street connectivity, the higher the probability of using walking or cycling as a mode of transportation [57,58], and the more reassured adolescents are about taking active modes of transportation to and from school [44,51].

#### 3.3.3. Built Environmental Quality

Objective built environmental quality: These studies focus on the impact of the comprehensive functional quality of the pedestrian environment and the functional quality of a single public open space. Five studies found that the higher the quality of the neighborhood walking environment, the easier it is to increase the probability of active travel by children [51], to increase the likelihood of active travel by adults [59], to increase levels of adult traffic walking or cycling [57], to increase recreational physical activity [60], and to increase moderate-to-high-intensity physical activity [41]. In addition, seven studies pointed out that the attractiveness, safety, convenience, maintenance, diversity, and high-quality characteristics of public open spaces, especially parks, significantly affect physical activity and lead to more leisure-time physical activity [22,32,35,50,61,62,63].

Subjective built environment quality: Based on exploring the impact of objective built environmental quality and the interaction between individual daily physical activities and built environmental elements, five studies incorporate individual subjective feelings into their analyses, focusing on perceived effects of the walkability, aesthetics, safety, and maintenance of the neighborhood built environment [45,52,58,62,64]. Three studies found a correlation between perception of the built environment and physical activity [33,34,44]. Two of the three studies showed that residents have stronger perceptions of walkability and aesthetics in their neighborhood or public open spaces and show higher levels of recreational activity and are more likely to achieve the recommended amount of physical activity [34,44]. In addition, 10 studies showed that the perception of safety factors related to crime and traffic might affect residents’ willingness to engage in outdoor activities, thereby affecting their traffic and leisure activity levels [31,42,43,45,46,47,48,49,55].

## 4. Discussion

This scoping review aims to analyze the state of the art in physical activity research, which includes the interaction between the built environment and the social environment and the impact of the combination of built and social environments on physical activity.

First, we discuss the impact of social and built environments on physical activity. Second, we discuss the relationship and interaction between social and built environments in physical activity research. Finally, we discuss the results in light of the potential advantages of interdisciplinary approaches for promoting physical activity.

### 4.1. Influence of Individual, Social, and Built Environmental Characteristics on Physical Activity

In this section, we further discuss the main result in detail. First, we have already noted that a large number of studies have focused on the adolescent population. In previous behavioral research, adolescence has been regarded as a critical period for behavioral change [65]. Furthermore, studies have shown that exercise habits developed during childhood and adolescence are highly likely to carry over into adulthood [66,67]. Therefore, promoting the habit of participating in physical activity in adolescence is particularly important in one’s life. This may explain researchers’ primary focus on physical activity research in adolescents.

Another research trend that we found is physical activity studies of females. The possible reason behind this is that research has documented that females are less physically active than males [68,69,70,71,72] and less likely to participate in physical activity outdoors due to safety concerns [73]. These barriers can potentially lead to inactivity and poor health outcomes. Therefore, females have been identified as a high-priority group for physical activity interventions [74].

In our selected articles, no studies focused on male groups alone. As some studies on men’s health behavior point out, a possible reason is that males have overall been underrepresented in physical activity intervention studies [75].

#### 4.1.1. Social Environment

In our review, the social environment was shown to strongly influence the physical activity of all age groups, in particular, aspects of social interaction, social support, and social cohesion.

Our review showed that in the adolescent studies, the social environment, especially aspects of parental values, parental constraints, and interaction between parents and adolescents, influences their physical activity pattern [36,37,42,52]. For example, a higher degree of family participation in physical activity is positively correlated with adolescent physical activity [36]. These findings align with previous research that identified family structure, parental values, educational level, and work situation indirectly affecting children’s physical activity behavior [76,77]. In line with this, previous research showed that having social interaction with peers can be a reason that adolescents are physically active [78]. Our review results confirm this by showing similar evidence that being able to hang out with friends and peers in the neighborhood can motivate adolescents’ physical activity behavior [33].

Another crucial social environmental factor for adolescents’ physical activity is social support. Social support has been considered a buffer against physical activity decline during the transitional period [78]. Our results show that verbal or active support for adolescents’ physical activity from family [37], friends [36], and peers [31,33,35] directly affects adolescents’ physical activity behavior. For example, adolescents who received more support for physical activity from friends participated in physical activity more often [36]. Four of the thirteen selected studies on adolescents indicate that friends and peers are the most influential source of social support for adolescent participation in physical activity [26,31,33,36]. This might be explained by the fact that adolescents spend most of their time in school, and adolescents in this age group gradually receive less parental supervision and more peer influence [73,79,80].

Social support is also an essential influencing factor for other demographic groups. For example, our results indicate that women who receive support from their families for physical activity are more likely to reach the recommended physical activity level compared to those who do not [46,47,48,49,55,64]. There is also evidence that social support may be more influential for women, especially the support they receive from their family [81,82,83]. Similar evidence was also found in a study with older people, in which the social environment was considered a more critical influencing factor of physical activity than the built environment [84]. For example, older people with neighbor/family member relations can take a walk, providing a better chance of achieving the recommended level of physical activity [40,45]. Previous studies are in line with these findings that social support from family, friends, and community can motivate positive physical activity behaviors in various demographic groups [85,86].

Social cohesion is generally defined as building shared values and making people feel engaged in common causes, facing challenges, and being members of the same community [87]. Our review showed that social cohesion impacts the physical activity of all demographic groups. For example, at the community level, higher social cohesion is associated with lower crime rates, and lower community crime rates tend to be associated with greater participation in physical activity [38,56,57,64].

#### 4.1.2. Built Environment

Our review shows that researchers have evaluated various built environmental factors. Accessibility and connectivity have received most of the attention. This might be because these two factors are directly measurable via geo-data, are easy to quantify and standardize, and because research results are easy to translate into policy [88]. However, GIS-driven geo-data also have limitations as objective geo-data cannot fully reflect the quality of the built environment [89].

In our review, it is shown that a number of researchers have started to investigate subjective perceptions of accessibility, connectivity, and built environmental quality. This might be because it was clear that the built environment’s impact on an individual’s physical activity behavior cannot be fully explained by objectively measured indicators [89,90]. For example, our findings show that perceived access was more important than objective measures of park access (tract-level park count, distance to nearest park, percent of tract covered by parks) for children and adolescents [36]. In addition, different resident groups may have different perceptions of the same environment and facilities [91]. For example, people with higher green space requirements and expectations usually think that greenery conditions in their neighborhood are bad, and they are concerned about outdoor activity experiences. In comparison, people with lower requirements and expectations may be more optimistic and believe that the greenery is of good quality [39]. Environments with the same objective measurement results can, thus, have different health effects on different groups.

### 4.2. Interaction and Relationship between Social and Built Environments

#### 4.2.1. Interaction

Although all selected studies included both social and built environments, only one study discussed the interaction between social and built environments. This study aimed to investigate whether social and built environmental interactions are associated with physical activity in underprivileged communities in the UK [62]. The research result suggests that the social environment moderates the built environment’s impact on physical activity. For example, the findings suggest that community cohesion and safety moderate the impact of physical barriers (e.g., the destruction of public spaces and buildings) on residents’ walking behavior, i.e., when residents perceive their community as having a higher level of integration. The damage to public spaces and buildings can affect residents’ physical activity. In addition, social interaction moderates the impact of aesthetics of the built environment on physical activity; for example, when residents have a higher level of social interaction, the aesthetics of the built environment affect individual physical activity behaviors. These findings suggest that aspects of the social environment may be more critical than physical aspects in encouraging individuals to be active in deprived environments [62].

As a concluding statement with regard to this topic, we can argue that community-level and individual-level socio-environmental factors influence individual physical activity outcomes and moderate the link between built environmental factors and physical activity. However, the studies included in this article only discuss the synergistic effects of social and built environments in a deprived neighborhood on residents’ physical activity. We also require more research and evidence across different community types to further define the interaction between society and the built environment. For example, in affluent and family-based communities, we need to examine whether the physical activity of residents in these communities is also affected by the synergistic effect of the social environment and the built environment.

#### 4.2.2. Relationship

**Figure 2:**
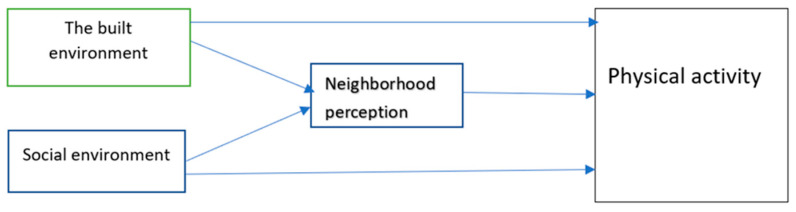
The relationship between built and social environments and neighborhood perception and their impacts on physical activity.

Regarding the relationship between social and built environments in physical activity research, we have found a collaborative relationship between these two environments in our review. We found that social and built environments both impact physical activity. Together, they form the neighborhood individuals are situated in, and after processing the information, the individual creates a neighborhood perception, which can also influence an individual’s PA pattern [31,58,62,64] (Figure 2).

Researchers have proposed that environmental perception is the psychological environment formed by the perceiver after receiving and processing information in the physical environment, which could guide external behavior [92]. For example, for residents who were satisfied with objective environmental quality and neighborhood safety, their perceptions of walkability and safety in their neighborhood or public open spaces were higher, and they were more likely to achieve the recommended amount of physical activity [58]. Furthermore, our selected studies of women showed a weak correlation between physical activity and the built environment compared to the perceived environment. In particular, the perception of good street lighting at night was a significant correlate of physical activity [43,47]. However, in contrast with this, some studies show that the aesthetic perception of the neighborhood environment is negatively related to the level of physical activity, especially with regard to walking [93,94]. Possible reasons for the inconsistent conclusions are that attractive neighborhoods with low mixed land use, functional inconvenience, and potentially poor street connectivity do not facilitate transportation-related physical activity for residents [95].

Generally, neighborhood perception as the collaborative result of social and built environments has become a new hotspot in physical activity research. Our findings show that neighborhood perception can help us capture specific groups’ concerns about specific social and built environmental contexts. For example, women are sensitive to the presence of street lights at night [43,46,47], parents are concerned about traffic safety [44], and older people are concerned about social interaction with community partners [40,45]. Through neighborhood perception, we can explore the influencing factors of physical activity from participants’ perspectives. Researchers have suggested that more research should focus on participants’ viewpoints of the environment [96]. However, there is still much to learn in this area, and further research is needed to deepen our understanding of the relationship between environmental perceptions and physical activity.

### 4.3. Potential Advantages of Interdisciplinary Approaches for Promoting Physical Activity

All 35 selected articles use social and built environments to understand an individual’s physical activity level. This shows that researchers are increasingly paying attention to the importance of studying and promoting PA from an interdisciplinary perspective. Our review suggests that social context can further explain physical activity behaviors in adolescents, women, older adults, and those from disadvantaged communities [31,33,35,42,45,46,47]. These findings are in line with the suggestion of previous studies that applying individual social environments to construct environmental analysis can better distinguish the influencing factors of physical activity among residents of different socioeconomic status, socio-demographic characteristics, and community types [97]. In addition to improving research accuracy, our review suggests that including measurement of the social environment in physical activity research may help tailor interventions to promote future physical activity. For example, the intervention design for women’s physical activity can start from their social environment; our result showed that participation in community activities is positively correlated with women’s physical activity [43,47,48,49,50,54]. Another example is deprived neighborhoods; our results demonstrate that social cohesion and interaction influence the built environment’s impact on an individual’s physical activity. Therefore, the design of physical activity interventions for poor communities should promote community participation in plan-making processes and increase the interaction between community residents [62].

### 4.4. Suggestions for Future Study

We have found a critical knowledge gap regarding the interaction of social and built environments. Our review found that social and built environments substantially impact an individual’s physical activity. In addition, researchers suggest that social and built environmental characteristics’ interactive effect may also impact an individual’s physical activity [97]. According to the social ecological model, human health is determined by individual factors, social networks, the built environment, and overall background conditions [98]. Furthermore, the social ecological approach to physical activity argues that individual characteristics, social environment, physical environment, and policies are all critical determinants of physical activity, which are interconnected and embedded in complex systems [99]. Therefore, understanding the interaction between these two factors may help us understand the synergy of social and built environments and utilize these two measures more effectively [100]. Therefore, we need more studies that analyze the interaction between social and built environments and the relevant impacts on physical activity.

The review result indicates that researchers fully recognize the importance of environmental perceptions in physical activity, and more literature has begun to take participants’ viewpoints on social and built environments as essential variables that impact physical activity [101,102,103]. Many recent studies applied neighborhood perception as the indicator, but in reality, perception elements are challenging to capture and require high accuracy [93]. To address this issue, researchers must continue to explore innovative methodologies and measurement tools that can provide a more accurate understanding of the impact of environmental perceptions on physical activity.

## 5. Conclusions

After conducting a thorough review of recent physical activity research, this scoping review has found evidence supporting the notion that both built and social environments can influence physical activity levels. The literature reviewed suggests that having access to safe and convenient infrastructure, as well as social support from friends and family, can increase the likelihood of engaging in regular physical activity.

Moving forward, it is essential for researchers to continue exploring participants’ perspectives on built and social environments in order to develop effective strategies to promote physical activity and improve public health outcomes. By combining insights from both built and social environments, policymakers and public health officials can develop more comprehensive and effective interventions to promote physical activity at the population level.

## Data Availability

Not available.

## Appendix A

**Table A1:** Overview of the characteristics of the selected studies.

Citation in APA	Research Aim	Social Environment Measurement	Built Environment Measurement	Physical Activity Measurement	Data Sample and Social Demographic
A. A. Eyler, 2003 [46]	The study goal was to determine personal, social environmental, and physical environmental correlates of physical activity among rural white women aged 20 to 50 years.	Three social environmental scales (social issues, social roles, and sense of community) were developed by summing the relevant series of questions.	Six questions that related to the built environment including site, traffic, presence of sidewalks, street lighting at night, places within walking distance, and places to exercise.	On the basis of self-reports about their physical activities per week, women were grouped into three categories that described their physical activity pattern: (1) Inactive (not engaging in any moderate or vigorous activities); (2) Insufficient (not meeting recommendations for either moderate or vigorous activities); (3) Meeting recommendations (engaging in moderate physical activity for at least 30 min five times per week or vigorous activity for at least 20 min three times per week).	A total of 1000 white women.
A. Carver et al., 2021 [37]	To examine how household/family characteristics, school-level social and physical environmental factors, and individual adolescent’s characteristics impact on their school-based PA during and after school hours in Hong Kong.	Household/family characteristics: Parents reported on several socio-demographic variables including their highest educational level. Socio-demographic variables of interest were number of motor vehicles at their household; household income per month; marital status; ethnicity/race, length of residence at current address; and number of children/adolescents in household. Neighborhood-level socioeconomic status (SES) was determined during sample selection and based on median household income for a specific TPU. Parental rules for physical activity: parents were asked whether (0 “no” or 1 “yes”) they had each of the 14 rules regarding their adolescent’s PA. Social support for physical activity from parents: Three items in the adolescents’ survey asked about parental support for PA.	Physical activity equipment at school: Adolescents were asked to report on whether (0 “no”; 1 “yes”) they had the following six pieces of sports equipment at their school: basketball hoops; soccer goal posts; balls; running/walking track; weight machines; indoor exercise machines (e.g., treadmills). Physical activity-friendly policy at school: PA-friendly policy at school was measured by two items that used a five-point scale to measure the adolescent’s perception of how often (0 “never” to 4 “always”) their school (1) offered supervised physical activities after school, and (2) allowed students to access playgrounds and sports fields after school hours. School physical activity-friendly index: A school PA-friendly index was computed by summing the standardized scores (z-scores) of PA equipment at school and PA-friendly policy at school.	Self-reported physical activity at school: Adolescents reported the frequency (days) and duration (minutes) of physical education classes per week at school, at two time-points, approximately six months apart. In addition, they reported on the frequency and duration of recess per week. Participation in afterschool sport: Adolescents reported how many sports teams or “afterschool” physical activity classes (not physical education) they participated in at school outside of standard school hours. Objectively measured moderate-to-vigorous physical activity at school and after school. The moderate-to-vigorous PA (MVPA) of a sub-sample of adolescents (N = 588) was objectively measured using the ActiGraph GTX3+ accelerometer (ActiGraph LLC, Pensacola, FL, USA) with data collected in 30 s epochs with a low-frequency filter. Parental leisure-time and transportation physical activity: Time (minutes/week) spent in parental leisure-time PA (LTPA) and transportation PA were each measured by items specific to those domains in the Chinese version of the International Physical Activity Questionnaire (Long form).	Cross-sectional study participants were Hong Kong adolescents attending secondary school, paired with their parent/caregiver (n = 1299 days).
A. D. M. Sawyer et al., 2017 [62]	This study aimed to explore independent and interactive effects of social and physical environmental factors on self-reported physical activity in income-deprived communities.	Responses to items assessing diverse aspects of the quality of the social environment, community cohesion, and social interaction were scored on 4–6-point Likert scales.	Selected items assessing the quality of the physical environment in terms of aesthetics, maintenance, and disorder were scored on 4-point Likert scales	Neighborhood walking was assessed through questions such as: “In a typical week, on how many days do you go for a walk around your neighbourhood?”. Participation in MPA was measured using the item: “In a typical week, on how many days do you do 30 min of moderate physical exercise such as brisk walking, cleaning the house–it doesn’t have to be 30 min all at once”	Participants were 5923 adults.
A. H. Auchincloss et al., 2019 [63]	The goal of this study was to test the hypothesis that a new greenway would result in an increase in moderate and vigorous levels of physical activity. A quasi-experimental pre–post paired location design was used to observe residents’ response to the new bicycle and pedestrian infrastructure.	Census tract data from the American Community Survey 2010–2014 describe area-level socio-demographics, housing, and transportation. Crime data: City of Philadelphia Police Department’s publicly available data	An environmental audit tool was based on the validated Path Environment Audit Tool (PEAT) (Troped et al., 2006) and focused on two domains that may deter or promote outdoor physical activity: “design and amenities” and “social disorder”.	System for Observing Play and Recreation in Communities (SOPARC) was used to scan each person passing through the observation area, to determine whether the person was engaged in MVPA (walking fast, bicycling, or running/jogging) or engaged in activity that was lower intensity (standing, sitting, walking slow/regular pace).	Pre-construction (2011) and post-construction (fall 2014), systematic observations (N = 8783), and environmental audit data were collected at the greenway and a comparison area. Post-construction intercept surveys were collected at the greenway (N = 175).
B. E. Ainsworth et al., 2003 [47]	The purpose of this study was to assess the relationship of personal, social, cultural, environmental, and policy variables with physical activity among women in ethnic minority groups.	Three social environmental scales (social issues, social roles, and sense of community) were developed by summing the relevant series of questions.	Six questions that related to the built environment which including site, traffic, presence of sidewalks, street lighting at night, places within walking distance, and places to exercise.	On the basis of self-reports about their physical activities per week, women were grouped into three categories that described their physical activity pattern: (1) Inactive (not engaging in any moderate or vigorous activities); (2) Insufficient (not meeting recommendations for either moderate or vigorous activities); (3) Meeting recommendations (engaging in moderate physical activity for at least 30 min five times per week or vigorous activity for at least 20 min three times per week).	A total of 917 African American women living in two counties in South Carolina.
B. Giles-Corti and R. J. Donovan, 2002 [32]	Spatial access to recreational facilities and perceptions of the neighborhood environment and physical activity levels were examined by the SES of area of residence.	SES	Geographic information systems (GIS) were used to develop indices for the eight main recreational facilities used by respondents (i.e., golf courses, gym/health club/exercise centers, sport and recreational centers, swimming pools, tennis courts, public open spaces, beaches, and rivers).	Physical activity level: the frequency and duration of physical activities undertaken in the previous 2 weeks (vigorous activity, light–moderate activity, walking for recreation, and walking for transport).	A cross-sectional survey of 1803 adults stratified by SES using a geography-based index was conducted.
B. Giles-Corti and R. J. Donovan., 2002 [22]	This project examined the relative influence of individual, social, environmental, and physical environmental determinants of recreational physical activity.	Social environmental: 1. Club membership; 2. Frequency of participation in physical activity by five significant others; 3. Frequency of a significant other doing physical activity with respondent.	Geo-coding: The spatial location of destination addresses (i.e., the recreational facilities) were determined using MapInfo. Road network analysis was undertaken to determine the distance between the origins and destinations, using the ArcInfo GIS software.	The frequency and total duration of all types of physical activity undertaken in the previous two weeks were collected: vigorous activity, light-to-moderate activity, walking for recreation, and walking for transport.	It involved a community survey of 1803 healthy workers and homemakers (aged 18–59 yrs).
B. Giles-Corti and R. J. Donovan., 2003 [50]	This study sought to examine individual, social environmental, and physical environmental correlates of walking.	Social environmental: 1. Club membership; 2. Frequency of participation in physical activity by five significant others; 3. Frequency of a significant other doing physical activity with respondent; 4. Dog ownership	Geo coding: The spatial location of destination addresses (i.e., the recreational facilities) were determined using MapInfo. Road network analysis was undertaken to determine the distance between the origins and destinations, using the ArcInfo GIS software.	The frequency and total duration of all types of physical activity undertaken in the previous two weeks were collected: vigorous activity, light-to-moderate activity, walking for recreation, and walking for transport.	The final sample included 1803 respondents from 277 districts (939 from the 80th percentile and above in terms of socioeconomic advantage and 874 from the 20th percentile or below).
B. K. Sanderson et al., 2003 [43]	The purposes of this study were to (1) describe the physical activity patterns among African American women aged 20 to 50 years living in rural Alabama; (2) compare the personal, social environmental, and physical environmental factors between more active and less active groups; and (3) discuss the implications of these findings for developing interventions designed to promote physical activity among African American women living in rural communities.	Three social environmental scales (social issues, social roles, and sense of community) were developed by summing the relevant series of questions.	Six questions that related to the built environment including site, traffic, presence of sidewalks, street lighting at night, places within walking distance, and places to exercise.	On the basis of self-reports about their physical activities per week, women were grouped into three categories that described their physical activity pattern: (1) Inactive (not engaging in any moderate or vigorous activities); (2) Insufficient (not meeting recommendations for either moderate or vigorous activities); (3) Meeting recommendations (engaging in moderate physical activity for at least 30 min five times per week or vigorous activity for at least 20 min three times per week).	A total of 567 African American women
C. C. Voorhees and D. R. Young., 2003 [48]	This study investigated the relationship between physical activity levels and socio-demographic, social environmental, and physical environmental factors.	Three social environmental scales (social issues, social roles, and sense of community) were developed by summing the relevant series of questions.	Six questions that related to the built environment including site, traffic, presence of sidewalks, street lighting at night, places within walking distance, and places to exercise.	On the basis of self-reports about their physical activities per week, women were grouped into three categories that described their physical activity pattern: (1) Inactive (not engaging in any moderate or vigorous activities); (2) Insufficient (not meeting recommendations for either moderate or vigorous activities); (3) Meeting recommendations (engaging in moderate physical activity for at least 30 min five times per week or vigorous activity for at least 20 min three times per week).	A cross-sectional, community-based convenience sample of 285 Hispanic/Latino women completed a face-to-face survey administered in Spanish.
Carroll-Scott et al., 2013 [39]	This study aimed to examine associations between built, socioeconomic, and social characteristics of a child’s residential environment on body mass index (BMI), diet, and physical activity.	Neighborhood social ties: —students answered three survey items: “How many of the grown-ups in your neighborhood do you know?”; “How many of the kids and teens do you know?”; and “Now think about your closest friends, do any of them live in your neighborhood?”. Neighborhood safety scale: this scale captured perceived safety in general, as well as neighborhood safety directly related to the ability to play or exercise outside. Neighborhood crime: two tract-level variables were created from the 2002–2010 aggregated incident codes consistent with previous criminology and health literature: total number of violent crimes and property crimes.	Perceptions of park access: students indicated their agreement (1 = strongly disagree, 5 = strongly agree) with the statement, “there are playgrounds, parks, or gyms close to my home or that I can get to easily”. Walking distance: distances between students’ homes to the nearest grocery store, convenience store, fast food restaurant, and park were calculated using 3 ArcGIS. Tract-level built environment: tract-level counts were calculated by summing the number of grocery stores, convenience stores, fast food restaurants, and parks within each tract and 20 m buffer.	Students answered the Patient-Centered Assessment and Counseling for Exercise (PACE) physical activity frequency item “In the past week, how many days did you exercise for at least 30 min (walking, playing, sports, gym class)”.	Participants were 1048 fifth- and sixth-grade students who completed school-based health surveys and physical measures in the fall of 2009. Student data were linked to the US Census, parks, retailers, and crime data. New Haven, The United States.
D. Benes-Nadworny, 2016 [35]	This study, guided by Stokols’ Social Ecological Model for Health Promotion, utilized data from the National Survey of Children’s Health (2011/12) and was designed to examine social environmental and physical environmental factors related to MVPA levels in early- and mid-adolescent Hispanic females (n = 1830).	Social environment level. Variables of the social environment were classified in three categories of support including peer, parental, and social.	Physical environment level: included attendance of safe schools, presence of neighborhood amenities, and presence of detracting neighborhood elements.	MVPA was measured using the question “How many days during the past week did your child exercise, play a sport or participate in physical activity for at least 20 min that made him/her sweat and breathe hard?”.	Data from 897 Hispanic female adolescents were included in this study.
D. Crawford et al., 2010 [36]	To determine the independent contributions of family and neighborhood environments to changes in youth physical activity and body mass index (BMI) z-score over 5 years	Home environment: parents completed a questionnaire 2 weeks (on average) before their child wore the accelerometer. The questionnaire collected demographic information on the child’s sex and age and on maternal education as a proxy for socioeconomic position (low ¼: some high school or less; medium ¼: high school or technical certificate, high-tertiary education), consistent with earlier studies; parental marital status (married/living as married, not married) and the number of children under the age of 18 years living in the household. Social support: at baseline, parents reported how often the father/male carer, mother/female carer, and siblings actively participated in physical activity with the child, and how often the father/male carer and mother/female carer provided support for physical activity such as taking them to training, providing money for participation, and buying sports clothing/equipment for their child (adapted from existing measures).	Home physical environment: parents were asked whether they had in their yard or garden a swimming pool/spa, trampoline, sandpit/swings/play equipment, or basketball ring and whether their child used nine types of physical activity equipment at home (recoded as “has item” or “does not have item”). Neighborhood environment. Aspects of the baseline neighborhood physical activity environment were objectively measured using a geographic information system (GIS) in 2004–2005. Spatial analyses were conducted using ESRIArc View 3.3 and related extensions. Destinations: using the GIS, the number of freely accessible public open spaces (no fees or restricted opening hours) and the number of public open spaces classified as sport/recreation within a 2 km radius of each participant’s residence were computed. Road connectivity: using the GIS, the number of intersections and cul-de-sacs within 2 km of each child’s home was computed, and the number of Xfour-way intersections was expressed as a proportion of the total number of intersections. Traffic exposure: using the GIS, the total length of “busy” roads (freeways, highways, or arterial roads) and the total length of “local” roads (identified using VicMap Transport, January 2004) were summed to provide an indicator of traffic exposure.	Children’s physical activity. At each of the three time points, children were asked to wear a Manufacturing Technology Inc (Actigraph Model, AM7164-2.2C, Fort Walton Beach, FL, USA) uniaxial accelerometer for an 8-day period during waking hours, except during bathing and aquatic activities. Parent’s physical activity: parents reported the total time in a typical week that they and their partner (if applicable) usually spent doing vigorous physical activity and moderate activity (for at least 10 min continuously), watching TV, playing electronic games, and using the computer during their leisure time.	A total of 301 children (10–12 years at baseline).
de Souza Andrade, A. C., et al., 2019 [58]	The aim of this study was to investigate the relationship between built and social environments and leisure-time physical activity (LTPA) among adults living in an urban center.	1. Individual: age (years), sex (male or female), current occupation (yes/no), marital status (with a partner: married/de facto relationship, or with no partner: single/divorced/widow), and time of residence (years). 2. The socioeconomic status (low, middle, or high, divided into tertiles) was measured at the individual level and built up based on the information on household goods consumption and the schooling of the head of the household.	Walking environment—sidewalk paving, trees for shading, sidewalk width at the smaller extremity, and favorable perception for walking. Spaces for physical activity and leisure—the presence of spaces and facilities for physical activity, the presence of parks and squares, and favorable perceptions of the physical activity environment. Aesthetic quality—political advertisement, presence of trees and gardens, cleanness of the environment, and presence of nuisance noise. Physical disorder—trash (needles, cigarettes, tin, and condoms) and presence of graffiti on public equipment. Safety—public lighting, safety items, and police surveillance. Services—grocery stores, public or private health facilities, and public or private gyms. Scale items were scored zero to five. High scores reflect a positive environmental assessment, with the exception of the physical disorder scale, to which the opposite interpretation applies	1. Physical activity was measured using the long version of the International Physical Activity Questionnaire; time spent on physical activities per week was assessed across different domains. 2. Leisure-time physical activity corresponded to the product of frequency (days/week) and average duration (minutes/day) of walking and mild, moderate, or vigorous activities (the latter multiplied by two). Only continuous activities lasting 10 min or more were considered.	This study comprised 3815 individuals living in 147 neighborhoods.
L. Haughton Mcneill et al., 2006 [54]	This study tested an explanatory model of physical activity hypothesizing direct and indirect effects of individual, social environmental, and physical environmental influences on physical activity.	Social support using a 5-point Likert scale that measured participants’ agreement or disagreement from 1 (strongly disagree) to 5 (strongly agree) with the following statements: “There are people in my life willing to do physical activity with me,” “I’d be more physically active if I had help with other responsibilities in my life,” “My family members are supportive of me being physically active,” “I’d be physically active if I had more information about it,” “My friends are supportive of being physically active,” and “I’d be active if my family or friends were more supportive.”	Participants were asked to rate the quality of their neighborhood regarding criminal activity, traffic, and pleasantness for engaging in physical activity using a 4-point Likert scale from 1 (very unsafe/unpleasant) to 4 (very safe/pleasant). To assess the availability of facilities, participants were asked if their neighborhood had walking/biking trails, parks, and outdoor/indoor places to exercise. Participants responded by answering yes or no.	Participants were asked to respond to two questions: How many days per week do you walk for at least 10 min at a time, and on days when you walk for at least 10 min at a time, how much total time do you spend walking? Response options for the first question ranged from 0 to 7 days per week, and response options for the second question ranged from 10 min to 60 or more min (in 10 min increments).	Participants were 1090 African American and white lower- and middle-income adults, recruited from the waiting rooms of two public health centers in St. Louis, Missouri, during a 3-month period in spring 2002.
E. L. Eyre et al., 2014 [42]	The aim was to examine environmental influences on children’s PA from a qualitative perspective in parents from low-SES wards in Coventry, UK.	Social environment was examined through: 1. Knowledge and beliefs about PA; 2. Home environment; 3. School environment; 4. Parental constraints and gender or age; 5. Parents’ life constraints.	Built environment-related questions include: 1. Distance to facilities; 2. Neighborhood safety; 3. Pleasant and aesthetic environment; 4. Cycle and walking networks.	Children’s physical activity was assessed using Instruments for Assessing Levels of Physical Activity and Fitness (ALPHA) environment questionnaire.	A total of 59 parents of children in year 4 (aged 8–9 years) completed the ALPHA environmental questionnaire. Sixteen of these parents took part in focus group discussions examining environmental facilitators and barriers to their child’s PA (March–April, 2013).
F. Ducheyne et al., 2012 [52]	this study examined individual, social, and physical environmental correlates of never and always cycling to/from school among 10- to 12-year-old Belgian children living within a 3.0 km distance from school.	Parents indicated agreement or disagreement with thirteen statements, which were either newly developed or adapted from existing scales, regarding their perceptions of their local neighborhood.	Physical environmental factors and route factors: the parent version of the “Neighborhood Environmental Walkability Survey for Youth” (NEWS-Y) was used to assess potential cycling to school-related environmental variables.	Parents indicated how many days a week their child (1) walked, (2) cycled, was (3) driven by car, or (4) used public transport to travel to and from school during fall, winter, and spring.	A total of 1235 parents (response rate = 80%) completed the questionnaire.
H. Chaudhury et al., 2016 [45]	This study examined the relationship of neighborhood physical and social environments with physical activity among older adults.	The social environment included membership in a sports group or recreational organization, and if the older adult had walked or engaged in physical activity with a neighbor in the past 12 months. The social nature of physical activity was also measured as the frequency in which a spouse, close family member, or friend participated in physical activity with the respondent in the past 12 months.	1. Perception of physical environment motivators; 2. Neighborhood walkability scale; 3. Perception of neighborhood safety due to crime and traffic; 4.The neighborhood safety scale; 5. Perception of neighborhood amenities and accessibility scale.	1. Physical activity level: participants provided information on their weekly physical activity levels when asked, “In a typical week, how many hours in total do you spend participating in physical activity?” 2.Physical activity type: participants were asked about the type of physical activity (or activities) they participated in during the previous four weeks. 3. Physical activity location: respondents were asked about the spaces or locations where they performed their physical activity or activities	A cross-sectional telephone survey was conducted with 434 older adults in eight neighborhoods in greater Vancouver, Canada, and Portland, United States.
J. Kirby and J. Inchley, 2013 [31]	The aim of this paper was to explore the broader context in which adolescent girls walk and to investigate their walking behaviors, experiences, and attitudes.	Family Friend/peers	Safety Aesthetics Proximity/access	Where they go walking When walking takes place How much walking Type/purpose of walking Walking histories	Focus group discussions and a mapping exercise were carried out with 27 adolescent girls from one urban and one rural school in Scotland.
J. L. Thompson et al., 2003 [49]	The purpose of this study was to determine the relationship among physical activity and various personal, social environmental, and physical environmental factors in Native American women.	Three social environmental scales (social issues, social roles, and sense of community) were developed by summing the relevant series of questions.	Six questions that related to the built environment including site, traffic, presence of sidewalks, street lighting at night, places within walking distance, and places to exercise.	On the basis of self-reports about their physical activities per week, women were grouped into three categories that described their physical activity pattern: (1) Inactive (not engaging in any moderate or vigorous activities); (2) Insufficient (not meeting recommendations for either moderate or vigorous activities); (3) Meeting recommendations (engaging in moderate physical activity for at least 30 min five times per week or vigorous activity for at least 20 min three times per week).	A total of 350 Native American women aged 20 to 50 years participated in the study.
J. Veitch et al., 2010 [33]	This study examined associations between individual, social, and physical environmental factors and the frequency with which children play in particular outdoor locations outside school hours. This study also investigated whether the frequency of playing in outdoor locations was associated with children’s overall physical activity levels.	Parents were asked how much they agreed or disagreed with the statements: “I do not have enough time to transport my child to activities”; “There is a high crime rate in my neighbourhood”; “It is safe for my child to play or hang out in the street outside our house”; “Stranger danger is a concern of mine”; “My child has many friends in this neighbourhood”; and “Lots of children play or hang out in the street outside our house”. Parents were also asked to report how often “As a family we go to the park”, with response options collapsed to at least once per week or less than once per week.	Parents were asked about their yard size with responses collapsed into two categories: no yard or small yard (e.g., unit); and medium or large yard (standard block of land or 1/4 acre or more). They also reported whether they lived on a main arterial or busy throughway for motor vehicles; and whether they lived on a cul-de-sac, court or no-through road. Finally, parents indicated how much they agreed or disagreed with the two statements “I am satisfied with the quality of parks in my neighbourhood” and “I am satisfied with the quality of playgrounds in my neighbourhood”.	Parents: survey items required parents to report how often their child played in the yard at home, their own street/court/footpath, and the park/playground outside school hours on weekdays and on weekend days during a typical week. Children: children were asked to wear an accelerometer (Manufacturing Technologies, Inc [MTI] Model 7164; Acti-graph, Inc, FL, USA) attached to an elasticized belt at hip level for eight consecutive days, removing it only for sleeping, showering, or swimming	Participants including 8–9 year old children and their parents (n = 187) were recruited from a selection of primary schools of varying socioeconomic status across metropolitan Melbourne.
J. Gao et al., 2015 [56]	This study aimed to examine the association between physical and social environments (both at individual and neighborhood levels) and LTPA among the Chinese elderly.	Social attributes of neighborhood social participation were assessed by asking respondents how often in the past 12 months they participated in eight different activities: (1) Visiting family or friends; (2) Recreational activities involving other people; (3) Physical and cultural activities in the neighborhood; (4) Attending a series of lectures in the neighborhood; (5) Self-management group, mutual-help group; (6) Volunteer or charity work; (7) Activities of political organizations or associations; (8) Dining out or shopping with other people. Social cohesion was assessed by the related module of Neighborhood Scales consisting of 4 items: (1) people around here are willing to help their neighbors; (2) people in my neighborhood generally get along with each other; (3) people in my neighborhood can be trusted; and (4) people in my neighborhood share the same values.	The physical attributes of neighborhoods in the current study and two modules of Neighborhood Scales were used to assess the aesthetic quality (AQ) and walkability of the neighborhood.	Leisure-time physical activity: the last 7-day weekly minutes of recreational walking and moderate and vigorous intensity physical activity were estimated using the Chinese long form of the International Physical Activity Questionnaire.	A total of 2783 elders were included in the study.
J. X. Fan et al., 2015 [59]	This research investigated participation rates in 3 modes (walking, biking, and walking to public transportation) of active commuting (AC) and their socio-demographic and physical environmental correlates in rural America.	1. Demographic variables: tract-level residents’ median age, percentage of Asian Americans, percentage of non-Hispanic blacks, percentage of Hispanics, percentage of foreign-born population, percentage of people who lived in college dorms, and percentage of people who lived in military quarters. 2. Socioeconomic variables included tract-level median household income (in $1000), median housing value (in $10,000), percentage of housing units that were owner-occupied, and percentage of residents 25+ with a college degree or higher. 3. Crime rate: number of crimes per 1000 persons at the county level 1999–2008.	The physical environment included the built environment described by the three neighborhood Ds: population density, destination diversity, and pedestrian-friendly design. Spatial data including census tracts and road networks were constructed from the data CD-ROMs distributed with ArcGIS 9.3 by the Environmental System Research Institute (ESRI) and the StreetMap USA file (a TIGER (Topologically Integrated Geographic Encoding and Referencing) 2000-based streets dataset enhanced by ESRI and Tele Atlas).	Three aggregate AC (active commuting) measures at the tract level: (1) percentage of workers 16 and over who walked to work, (2) percentage of workers 16 and over who biked to work, and (3) percentage of workers 16 and over who took public transportation to work.	Decennial Census, 2000.
K. A. Morris et al., 2020 [64]	This study aimed to longitudinally examine the social ecological factors associated with physical activity and screen time amongst mothers living in socioeconomically disadvantaged neighborhoods, and whether these differed according to their child’s age.	Thirteen independent variables encompassing three constructs of the social ecological model (i.e., intrapersonal, social, physical environmental) were included in the analysis. Social factors: 1. Social support from family/spouse; 2. Social support from friends/work colleagues; 3. Childcare.	Thirteen independent variables encompassing three constructs of the social ecological model (i.e., intrapersonal, social, physical environmental) were included in the analysis. Physical environment factors: 1. Neighborhood walkability; 2. Neighborhood aesthetics; 3. Personal safety; 4. Neighborhood cohesion; 5. Number of televisions per household.	Leisure- time and transport-related physical activity: participants reported their frequency and duration of physical activity over the past week across four domains (occupation, transport, leisure, domestic) using the International Physical Activity Questionnaire—long form (IPAQ-L).	Data were from 895 mothers living in socioeconomically disadvantaged neighborhoods (mean age 36.7 years) at baseline and three-year follow-up.
K. R. Evenson et al., 2003 [55]	The objective of this study was to describe physical activity and the personal, social environmental, and physical environmental correlates for Latina immigrants.	Three social environmental scales (social issues, social roles, and sense of community) were developed by summing the relevant series of questions.	Six questions that related to the built environment including site, traffic, presence of sidewalks, street lighting at night, places within walking distance, and places to exercise.	On the basis of self-reports about their physical activities per week, women were grouped into three categories that described their physical activity pattern: (1) Inactive (not engaging in any moderate or vigorous activities); (2) Insufficient (not meeting recommendations for either moderate or vigorous activities); (3) Meeting recommendations (engaging in moderate physical activity for at least 30 min five times per week or vigorous activity for at least 20 min three times per week).	A total of 671 first-generation Latina immigrants aged 20 to 50 years who were living in North Carolina.
L. Franzini, 2009 [44]	We investigated the association between physical and social neighborhood environments and fifth-grade students’ physical activity and obesity.	1.Social Cohesion (5 items): to assess closeness, common values, trust, and helpfulness at the community level; 2. Informal Social Control (5 items): to assess willingness to intervene if children misbehaved or skipped school or if a community problem arose.	1. The Traffic Scale (2 items) measured the flow of traffic and the number of lanes on the face block; 2.The Physical Disorder Scale (6 items) assessed the frequency of abandoned cars, litter, and graffiti; 3.The Residential Density: scale (1 item) measured the prevalence of residential units that were not stand-alone houses or duplexes; 4: Mixed Land Use Scale (1 item) assessed whether the face-block was primarily residential (these scales were recoded).	(1) The number of days in the past week of vigorous exercise (makes the heart beat fast or the child breathe hard) for at least 20 min; (2) The number of days in the past week of moderate exercise (did not make the heart beat fast or the child breathe hard) for at least 30 min; (3) The number of days per week of physical education or gym class at school; (4) The number of sports teams in which the child participated during the past 12 months; (5) Participation in other organized physical activity or lessons (e.g., karate, dance, gymnastics, tennis).	A total of 650 fifth-grade students and 1 of their primary caregivers (usually a parent).
M. Gascon et al., 2019 [38]	Within the framework of the European Physical Activity through Sustainable Transport Approaches (PASTA) project, we aimed to explore the correlates of walking for travel in European cities.	Social norms and mobility culture in the neighborhood: Three different questions were used to determine the community context of each individual with regard to walking (Götschi et al., 2017): (a) “Most people who are important to me think that I should walk for travel,” (b) “In my neighborhood walking is well regarded,” and (c) “In my neighborhood it is common for people to walk for travel.” Response options were on a 5-point Likert-type scale with 1 for “very much disagree” to 5 for “very much agree.”	Built environmental characteristics: the same built environmental characteristics were included in the present analysis for both the residential and the work/study addresses, using a 300 m radial buffer.	Physical activity: minutes of walking per week for travel, which was the result of combining the GPAQ questions “In a typical week, on how many days do you walk for at least 10 min continuously to get to and from places?” and “Typically, how much time do you spend walking on such a day?”	A total of 7875 participants were included in the main analyses. Out of those, 6957 participants also provided work or study addresses and were included in the secondary analyses.
N. H. H. D. Trang et al., 2012 [51]	This paper aimed to describe the changes in the prevalence of active commuting to and from school, and to prospectively examine the predictors of active commuting among adolescents from Ho Chi Minh City (HCMC).	Household economic status was assessed through questions on ownership of 14 assets, which were used to construct a household wealth index by assigning a weight to each asset.	The direct distance between school and home was calculated using the geographic coordinates of each participant’s home, and those for their schools were measured by project staff using GPS devices (Garmin E-trex^®^). Neighborhood environment: parents were asked about their concerns regarding traffic safety in their neighborhoods and the presence and quality of sidewalks.	Commuting to Schools: participants were asked if they were driven, took a bus/minibus, motor-biked, or biked or walked to and from school every weekday, using a validated youth Physical Activity Questionnaire (PAQ), which was adapted for use in Vietnam and validated for adolescents in HCMC.	A representative sample of 759 adolescents from 18 schools in HCMC.
R. G. Prins., 2019 [40]	Evaluated the effects of a small-scale physical environmental intervention (designated walking route), a social environmental intervention (neighborhood walking group) and the combination of both on walking behavior of older adults living in deprived neighborhoods.	Social environmental intervention	Physical environmental intervention	Recreational walking and utilitarian walking were measured with the long version of the International Physical Activity Questionnaire. Participants reported the average time per day they engaged in recreational walking and utilitarian walking over the past week, and the numbers of days per week they engaged in these activities.	Survey data of 644 older adults residing in four deprived neighborhoods of Rotterdam, the Netherlands.
R. Hoekman et al., 2017 [41]	This study investigated the intensity of sports participation in the Netherlands comparing urban and rural areas.	1. Social environment: zip code of the neighborhood; 2. Socioeconomic status scores of the neighborhoods were based on an aggregate indicator of educational level, position in the labor market, and income level of neighborhood residents; 3. Neighborhood safety was obtained by aggregating information from the “Level of Living Barometer” (Van der Reijden, Van Woerkens, Leidelmeijer, Marlet, & Schulenberg, 2013).	Physical environmental measures were obtained from the Facility Monitor Sport (FMS). The reputed FMS provides geographical information on (nearly) all sport facilities in the Netherlands (more than 14,000).	Demographic information identified the respondent’s home address and length of residency, age, race/ethnicity, education level, and income level. Physical activity was measured using the 2001 Behavioral Risk Factor Surveillance System (BRFSS) physical activity module	A total of 17,910 Dutch inhabitants between 6 and 79 years of age.
S. D’Haese et al., 2014 [61]	The aim of this study was to investigate the association between objective walkability and different domains of children’s physical activity, and to investigate the moderating effect of neighborhood socioeconomic status in this relation.	Neighborhood SES: median annual household income data (National Institute of Statistics–Belgium, 2008) were used to determine neighborhood SES of the different statistical sectors. Neighborhoods were characterized as low SES (income < €22,359) or high SES (income ≥ €22,359) based on the median.	Residential density, intersection density, and land-use mix diversity were determined and z-scores were calculated. Walkability was calculated as follows: walkability = (2 × z-connectivity) + (z-residential density) + (z-land use mix). Objective neighborhood walkability of all statistical sectors was calculated using a geographical information system database.	Children’s self-reported PA was measured with the Flemish Physical Activity Questionnaire (FPAQ). Parents were asked to fill out the questionnaire at home together with their children and to report their child’s PA levels in a usual week. Objective PA was determined by accelerometers. Children wore an Actigraph™GT1M, GT3X or GT3X + accelerometer (15 s epoch) during waking hours for 7 consecutive days.	Children (9–12 years old; n = 606) were recruited from 18 elementary schools in Ghent.
S. M. P. L. Gerards et al., 2021 [53]	The aims of this longitudinal study were to investigate whether the physical environment and parenting practices have an impact on changes in children’s weekday time spent at various PA levels and whether associations between physical neighborhood environment and changes in children’s PA are moderated by parenting practices.	Social environment: parenting practices were measured using a validated questionnaire developed and validated by Davison et al.	Built environment: The SPACE checklist was used to assess physical neighborhood characteristics.	Children’s PA and SB levels were measured using accelerometers (ActiGraph, GT3X+, 30 Hz).	In total, 240 children aged 8–12 years were included in the analyses.
T. G. Cavazzatto et al., 2020 [60]	This study calculated the exposure–response rates of social ecological correlates of practicing regular (>150 min/week) leisure-time physical activity (PA) in 393,648 adults from the 27 Brazilian state capitals who participated in a national survey between 2006 and 2016.	Social: crime mortality (1/100,000 inhabitants), number of employees of physical activity-related companies (inhab. rate), family income < 1/2 min wage (%), family income from 1/2 to 1 min wage (%), family income from 1 to 2 min wage (%), family income > 2 min wages (%), percentage of women (%), life expectancy for men, women and in general (years), population (millions), and traffic accident mortality (1/100,000 inhabitants).	Environment and Transport: bus fleet/100,000 inhabitants, car fleet/100,000 inhabitants, PA companies (e.g., sports and recreational clubs, gyms) (inhabitants’ rate), and vehicle fleet/100,000 inhabitants (all type).	Physical activity levels were based on the following questions: “(1) In the last three months during your leisure-time, did you perform any exercise or sport?”; “(2) What is the main type of exercise or sport that you practiced?”; “(3) How many days a week do you usually practice exercise or sport?”; “(4) On these days, how long (minutes/day) do you perform the exercise or sport for?”.	A total of 572,477 participants from 27 Brazilian state capitals were included.
T. Ståhl et al., 2001 [34]	This study examines the relationships between reported physical activity, and the extent of perceived support for physical activity in physical and policy environments (e.g., facilities, programs, and other opportunities), and in the social environment.	Social environment: ten items measured perceived motivation to participate in sports and physical activity from family and friends, as well as less direct social influences such as newspapers, TV, workplace, school, community, politicians, doctor, and health insurance.	Supportive physical and policy environment: perception.	Physical activity: Respondents’ physical activity was assessed by one very general question: “Do you do any gymnastics, physical activity or sports?” The measure distinguished active people from inactive people, since the respondents answered either yes (1) or no (0). Interviewers were advised to explain that physical activity is defined in a very broad sense including, e.g., physically active commuting to work, gardening, competitive sport, etc.	In total, 3342 adults, 18 years or older, from six countries (Belgium, Finland, Germany, The Netherlands, Spain, Switzerland) were interviewed via telephone.
X. Zhu et al., 2014 [57]	This study examined changes in residents’ physical activities, social interactions, and neighborhood cohesion after they moved to a walkable community in Austin, Texas.	Positive social interactions were measured by the frequency of specific interactions; neighborhood cohesion was measured using a 5-point Likert scale, by asking the respondent how much he/she agreed or disagreed with relevant statements	The walkability for each respondent’s pre-move neighborhood was measured using the publicly available Walk Score (WalkScore.com, 2014), which captures environmental factors such as density of retail destinations, street intersections, and residential land uses.	Physical activities were captured by the number of days per week with ≥30 daily min of moderate physical activities and by frequencies (days/week and min/day) of specific activities.	Retrospective surveys (N = 449) were administered in 2013–2014 to collect pre- and post-move data about the outcome variables and relevant personal, social, and physical environmental factors.
